# Systematic review of clinical trials of cervical manipulation: control group procedures and pain outcomes

**DOI:** 10.1186/2045-709X-19-3

**Published:** 2011-01-11

**Authors:** Howard Vernon, Aaron Puhl, Christine Reinhart

**Affiliations:** 1 Canadian Memorial Chiropractic College 6100 Leslie St., Toronto, Ontario, M2H 3J1, Canada

## Abstract

**Objective:**

To characterize the types of control procedures used in controlled clinical trials of cervical spine manipulation and to evaluate the outcomes obtained by subjects in control groups so as to improve the quality of future clinical trials

**Methods:**

A search of relevant clinical trials was performed in PubMed 1966-May 2010 with the following key words: "Chiropractic"[Mesh] OR "Manipulation, Spinal"[Mesh]) AND "Clinical Trial "[Publication Type]. Reference lists from these trials were searched for any additional trials. The reference lists of two prior studies, one review and one original study were also searched. Accepted reports were then rated for quality by 2 reviewers using the PEDro scale. Studies achieving a score of >50% were included for data extraction and analysis. Intra-group change scores on pain outcomes were obtained. For determining clinically important outcomes, a threshold of 20% improvement was used where continuous data were available; otherwise, an effect size of 0.30 was employed

**Results:**

The PubMed search yielded 753 citations of which 13 were selected. Eight (8) other studies were identified by reviewing two systematic reviews and through reference searches. All studies scored >50% on the PEDro scale. There were 9 multi-session studies and 12 single-session studies. The most commonly used control procedure was "manual contact/no thrust". Four (4) studies used a placebo-control (patient blinded). For two of these studies with VAS data, the average change reported was 4.5 mm. For the other control procedures, variable results were obtained. No clinically important changes were reported in 57% of the paired comparisons, while, in 43% of these, changes which would be considered clinically important were obtained in the control groups. Only 15% of trials reported on post-intervention group registration.

**Conclusions:**

Most control procedures in cervical manipulation trials result in small clinical changes, although larger changes are observed in 47% of paired comparisons. The vast majority of studies do not result in subject blinding; the effect of unmasking of control subjects in these studies makes the interpretation of the existing clinical trials challenging. The greatest majority of trials do not report on post-intervention blinding. A small number of candidate procedures for effective control interventions exist. Much more research is required to improve this important aspect of clinical trial methodology in cervical manipulation studies.

## Introduction

Clinical trials of spinal manipulation for neck pain have been published since the early 1980's. Numerous reviews of these trials have been published in the ensuing years [1-3]. The lack of a valid control group has been a consistent criticism of this body of studies [1-5]. In 2005, Vernon et al. [6] reported on a candidate manoeuvre for a cervical sham manipulation (sham cervical thrust using a "drop" headpiece). In a small group of neck pain patients, 60% mis-registered the sham manoeuvre as a "real treatment". In these subjects, no clinically important changes were obtained post-intervention in paraspinal pressure pain thresholds (R-PPT decreased by an average of 1.2%; L-PPT decreased by an average of 6%) as well as in cervical ranges of motion.

In that report, the literature on studies of manipulation with sham/placebo manoeuvres was briefly reviewed. Of note were the studies of Hawk and her colleagues [7-9] who identified numerous issues attendant with the development and use of sham manipulations. Their work was focused on the lumbar spine and low back pain patients. The review by Ernst and Harkness [4] was also mentioned as one of the works critical of the extant clinical trials in manipulation for neck pain.

Vernon et al. [10] conducted a systematic review of the outcome of control groups used in clinical trials of conservative treatments for chronic neck pain. These trials included primarily laser and acupuncture studies; no study of manual therapy was included. In this review, the mean [95% CI] effect size of change in pain ratings in the no-treatment control studies at outcome points up to 10 weeks was 0.18 [-0.05, 0.41] and for outcomes from 12-52 weeks it was 0.4 [0.12, 0.68]. In the placebo control groups it was 0.50 [0.10, 0.90] at up to 10 weeks and 0.33. [-1.97, 2.66] at 12-24 weeks. None of the comparisons between the no-treatment and placebo groups were statistically significant. It was concluded that changes in pain scores in subjects with chronic neck pain not due to whiplash who are enrolled in no-treatment and placebo control groups were similarly small and not significantly different. As well, they do not appear to increase over longer-term follow-up. The placebo and no-treatment control procedures in these trials appeared to be successful in inducing relatively little therapeutic benefit.

There has been no similar review of the control procedures and control group outcomes of trials of manipulation in the cervical spine for neck pain and headaches, although a review of control group outcomes in lumbar spine trials has recently been published [11]. Such a review would assist clinicians and researchers in determining the validity of the existing evidence base as well as the applicability and generalizability of the control procedures which have been employed to date. It would also identify issues for consideration by future clinical trial groups.

## Methods

### Search Strategy

A search for randomized controlled clinical trials was performed in PubMed 1966-May 2010 with the following key words: "Chiropractic"[Mesh] OR "Manipulation, Spinal"[Mesh]) AND "Clinical Trial "[Publication Type]. Reference lists from these selected trials were searched for any additional trials. The reference lists of two prior studies, one review [4] and one original study [6] were also reviewed. Finally, after reviewing the retrieval lists from these searches, the authors identified some additional trials from the general literature.

### Inclusion Criteria

Studies were included into the quality review round if they fulfilled the following criteria:

a) randomized clinical trial

b) cervical spinal manipulation was the index treatment (studies of thoracic manipulation were excluded)

c) a control group was used in any of the following forms

a. placebo treatment

b. non-blinded control treatment

c. no-treatment or waiting list control

d) the clinical complaint was neck pain, neck and arm pain or headaches

e) data from a pain-related outcome was provided for each group at relevant times

e) English language

### Study Selection

The inclusion criteria were applied by the senior author to the titles and abstracts of the studies identified in the searches.

### Quality reviewing

Studies included in the review were then subjected to quality rating by two independent raters (not the senior author). Ratings were derived using the PEDro Scale [12] for a score out of 11. Scores were converted into a percentage figure. Each rater conducted a separate rating. After this, ratings were compared. When exact agreement was not achieved, a consensus method was used to resolve any disagreements in ratings. This method involved the two raters working together first. If any disagreements could not be resolved between them, the senior author joined the discussion and forced a consensus rating. Studies scoring higher than 50% were included in the review.

### Categorization of the included studies

Studies were separated into two categories: 1) single and 2) multiple intervention session trials.

### Data extraction and analyses

Data were extracted by a single author. The following data was extracted: complaint type, number of subjects in the control group, control intervention type, type of primary outcome measure, whether blinding was checked post-intervention, primary pain-related outcome data for the control group(s) (typically a VAS: means, variance measures, effect sizes). For determining clinically important changes, several criteria were employed. Where continuous data were available, a threshold of 20% improvement was used; otherwise, an effect size of 0.30 was employed [similar to Vernon et al. [10,13,14]. Data were not formally pooled, although, when possible, means (sd) of the outcomes of selected groups of trials were computed. Data from the index treatment group was not analyzed.

## Results

The PubMed search yielded 753 citations of which 13 were selected [15-26]. The manual search of Ernst and Harkness' study [4] identified no additional studies. The search of Vernon et al. [6] identified 3 additional studies [27-29]. The senior author identified 5 additional studies [30-34] for a total of 21 studies. There were four exceptions to the inclusion rules as follows: the trial by Bakris et al [33] was accepted for its unique approach to creating a control procedure; the trials by Buchman et al. [17], Tuttle et al. [23] and Dunning and Rushton [24] were accepted as manual control studies, even though the outcome measure was not pain-related.

The types of control interventions are described in Table 1. The quality scores and data extraction for these 21 studies are depicted in Table 2. All studies scored above 50% and were included in the review. The mean quality score for all these studies was 77.8 (11.7) %. There were 9 multiple session studies [15,16,27-29,31,33,34] whose average quality score was 82.5% (9.2). Of these, 6 were for headaches [15,28,29,31,34,35], 2 were for neck pain [16,27] and 1 was for another complaint (hypertension [33]). There were 12 single session studies whose average quality score was 74.2% (11.9). Of these, 10 were for neck pain and 2 were for other complaints in the upper limb. There was a statistically significant difference in the quality scores favouring the multi-session studies (t = 2.49, p = 0.01). Both groups of studies had an average of 24 subjects per group (range for multi-session = 9-40; range for single session = 8-54).

**Table 1:** Types of control interventions

Intervention type	Multiple session studies	Single session studies
Placebo: drug only	1	0

Placebo: non-manual therapy	1	1

Placebo: drug + sham manual therapy	1	0

Manual thrust at alternate site (randomly different [30], contralateral [32], ineffective site [33])	1	2

Manual contact: no thrust	2	10

Low level manual therapy	1	0

Low level non-manual therapy only	0	0

Low level manual + non-manual therapy	1	0

Waiting list	1	0

Figures add up to 22 due to use of 2 control groups in [Pikula]

**Table 2:** Review of studies of manipulation and control or placebo comparison

STUDY (First author name)	PEDro	REGION/COMPLAINT	"PLACEBO" MANEUVER	N (CONTROL GROUP)	BLINDING CHECKED?	PRIMARY OUTCOME MEASURE	PRIMARY OUTCOME FOR CONTROL GROUP
**Multi-session**							

Sloop, 1982 [27]	**8/11**	Neck pain	Diazepam (anamnestic) with no treatment	18	Yes	For neck pain: - VAS	VAS change @ 3 wks = 5 mm (±32 mm)
						- NVS	NVS = 28% subjects reported treatment helped

Nilsson et al., 1997 [28]	**9/11**	Cervical: headache	Low power laser light and deep friction massage	25	NR	- headache hrs	Avg HA (hrs/day ± inter4tile range) pre = 4.0; post = 2.4; Δ = -1.6 ± 2.5
						- headache intensity	Avg HA intensity (mm ± inter4tile range) Pre = 41; post = 36; Δ = -4.2 ± 26
						- analgesic use	Avg analgesics/day ± inter4tile range Pre = 1.0; post = 0.7; Δ = -0.3 ± 1

Bove and Nilsson, 1998 [29]	**9/11**	Cervical spine: Headache	Low power laser light and deep friction massage (described as both an "active control" and as the "placebo" group)	37	NR	pre = 2 wks post = 7 wks follow-up = 19 wks	
						-headache hrs	Avg HA hrs/day (95% CI) Pre = 3.4 (2.4-4.4) post = 1.9 (0.9-2.9); Δ = -1.5 follow-up = 2.2 (1.2-3.2); Δ = -1.2
						-headache intensity (VAS)	Avg HA intensity (95% CI) Pre = 37 mm (33-41) post = 34 mm (26-38); Δ = -3 follow-up = 26 mm (20-32); Δ = -11 *
						-analgesic use	Avg analgesics/day (95% CI) Pre = 0.82 (0.5-1.14) post = 0.59 (0-1.49); Δ = -0.23 follow-up = 0.56 (0.22-0.9); Δ = -0.26

Tuchin et al., 2000 [15]	**7/11**	Cervical spine - migraines	De-tuned interferential therapy	40	NR	Headaches per month	Avg HA/month (SD) Pre = 7.3 (6.53) post = 6.9 (6.6); ES = 0.06
						Headache intensity (VAS)	Avg HA intensity VAS (SD) Pre = 7.89 (1.2) post = 6.2 (1.7); ES = 1.17 *
						Headache duration (hrs) analgesics/month	Avg HA duration in hrs/episode (SD) Pre = 22.6 (27.4) post = 19.8 (17.7); ES = 0.12 Avg analgesics/month (SD) Pre = 20.1 (28.4) post = 16.2 (12.4); ES = 0.19

Alison et al., 2002 [16]	**8/11**	Neck and arm pain	Waiting list	10	N/A	-SF-MPQ	Median SF-MPQ (inter4ile range) Pre = 10.0 (9.0)
						- NPQ - VAS	Post = 7.5 (4.0); Δ = -2.5 * median NPQ (inter4tile range) Pre = 12.5 (4.0) Post = 11.5 (6.0); Δ = -1.0 median VAS (inter4tile range) Pre = 3.3 (3.5) Post = 3.8 (3.9); Δ = 0.5

Bakris et al., 2007 [33]	**9/11**	Cervical spine - hypertension	Manual contact, Inappropriate direction of thrust	25	NR	BP & pulse	Systolic BP: Pre = 145.3; post = 142.1; Δ = -3.2 Diastolic BP: Pre = 91.0; post = 89.2; Δ = -1.8 Pulse rate Pre = 73.3; post = 73.8, Δ = -0.5

Vernon et al., 2009 [31]	**9/11**	Cervical spine: headache	Sham manipulation with head thrust, but no segmental thrust + placebo meds	9	Yes^##^	Reduction of headache days	Outcome not reported for sham manipulation only

Borusiak et al., 2009 [34]	**8/11**	Cervical spine: pediatric headache	Light touch/no thrust	28	Yes	- % of days with HA	% of days with HA (SD) Pre = 41.2 (28.5) Post = 31.8 (28.3); ES = 0.33 *
						- Duration of HA	Duration of HA in hours (SD) Pre = 113.8 (115.1) Post = 107.2 (121.1); ES = 0.06
						- Intensity of HA	Intensity of HA VAS (SD) Pre = 4.9 (1.8) Post = 5.0 (1.8); ES = 0.06

Haas et al., 2010 [35]	**11/11**	Cervical spine/cervicogenic headache	Heat + light massage:		NR	Numerical rating scales for: A - Headache intensity	
			Group 1- 8 sessions	20			Percentage score - Group 1: Pre = 56.8 (15.8) 12 wk = 42.0 (20.6); ES = 0.81 * 24 wk = 41.5 (18.2); ES = 0.90 *
			Group 2- 16 sessions	20		B - Neck pain	Percentage score - Group 2: Pre = 58.7 (17.1) 12 wk = 49.4 (19.0); ES = 0.52 * 24 wk = 48.6 (21.4); ES = 0.52 *
							Percentage score - Group 1: Pre = 60.5 (21.4) 12 wk = 47.1 (24.2); ES = 0.59 * 24 wk = 47.2 (21.8); ES = 0.58 * Percentage score - Group 2: Pre = 48.5 (23.6) 12 wk = 42.8 (21.6); ES = 0.25 24 wk = 48.4 (23.1); ES = 0.004

**Single session**							

Pikula, 1999 [32]	**6/11**	Neck pain	1. SMT- contralateral 2. Detuned US	1-12 2-12	NR	VAS neck pain	1. Contralateral manip VAS (SD) Pre = 44.1 (27.5) post = 41.4 (28.4) ES = 0.10
							2. Placebo UltraSound VAS (SD) Pre = 50.4 (22.5) post = 46.5 (21.8) ES = 0.18

Haas et al 2003 [30]	**11/11**	Neck pain	Alternate site manipulation	52	NR	Pain (VAS) Measured immediately after and later that evening (~ 6 hours)	Pain VAS (SD) Pre = 40.4 (20.9) post = 24.7 (19.5); ES = 0.78 * follow-up = 28.7 (19.6); ES = 0.58 *

Buchmann et al., 2005 [17]	**6/11**	Cervical spine	Manual contact, no rotation, no thrust	8	NR	Dysfunctional motion segments	Pre contact = 13 dysfunctional segments
							Post contact = 13 dysfunctional segments

Martinez-Segura et al., 2006 [18]	**8/11**	Cervical spine - Neck pain	Manual contact, cervical rotation, no thrust	37	NR	Resting neck pain (VAS)	Resting neck pain VAS (SD) Pre = 5.5 (1.7) post = 5.1 (1.9) ES = 0.22

Fernandez De Las Penas et al., 2007 [19]	**7/11**	Cervical spine - healthy subjects	Manual contact, cervical rotation, no thrust	15	NR	PPT at elbow both ipsi and contral.	Ipsi elbow PPT Pre = 2.3(0.4); post = 2.3 (0.5); ES = 0 Contra elbow PPT Pre = 2.3(0.5); post = 2.3 (0.6); ES = 0

Ruiz-Saez et al., 2007 [20]	**8/11**	Cervical spine - trapezius MTrP's	Manual contact, cervical rotation, no thrust	36	NR	PPT at trapezius trigger points	Pre = 1.34 (0.4) Post = 1.27 (0.4); ES = -0.18 Post 5 min = 1.15 (0.4); ES = 0.48 * Post 10 min = 1.1 (0.5); ES = 0.53 *

Fernandez-Carnero et al., 2008 [21]	**8/11**	Cervical spine - tennis elbow	Manual contact, cervical rotation, no thrust	10	NR	- PPT	Affected elbow PPT Pre = 314.4 (11.6); post = 327.7 (18.6) ES = 0.88 * Contralateral elbow PPT Pre = 475.2 (78.5); post = 481.2 (84.6) ES = 0.07
						- Thermal pain threshold (TPT)	Affected elbow TPT (^o^C) Pre = 41.1 (3.4); post = 41.8 (1.3) ES = 0.3 Contralateral elbow TPT (^o^C) Pre = 44.3 (1.5); post = 43.4 (0.9) ES = 0.75
						- Pain free grip strength (PFG)	PFG (KG) affected side Pre = 14.7 (6.0); post = 13.6 (6.2) ES = 0.18

Fernandez De Las Penas et al., 2008 [22]	**8/11**	Cervical spine- healthy subjects	Manual contact, cervical rotation, no thrust	10	NR	C5-C6 Z-joint tenderness (PPT)	Left Z-joint PPT Pre = 316.4 (30.5); post = 311.8 (32.8) ES = 0.15 Right Z-joint PPT Pre = 315.0 (43.8); post = 312.3 (47.7) ES = 0.06

Tuttle et al., 2008 [23]	**7/10**^**1**^	Cervical spine - neck pain	Mobilization applied to non-symptomatic level	20	NR	AROM stiffness	NS decrease in flex/ext AROM NS increase in lat flex and rotation NS decrease in stiffness (data not reported; only graphic data)

Dunning and Rushton, 2009 [24]	**6/10**^**1**^	Cervical spine - EMG of biceps muscle	Manual contact, cervical rotation, no thrust	54	NR	Biceps resting EMG	21.12% (±5%) increase in resting EMG of right bicep after sham
							17.15% (±7%) increase in resting EMG of left bicep after sham

Mansila-Ferragut et al., 2009 [25]	**8/11**	Upper cervical spine - neck pain	Manual contact, cervical rotation, no thrust	19	NR	- PPT at the sphenoid	PPT (95% CI) Pre = 0.8 (0.7 - 0.9) Post = 0.7 (0.5 - 0.9); Δ = -0.1
						- Active mouth opening	Active mouth opening in mm (95% CI) Pre = 36.2 (34.3 - 38.2) Post = 35.9 (33.7 - 38.0); Δ = -0.3

Sterling et al., 2010 [26]	**8/11**	Neck pain findings	Manual contact	17	NR	- PPT at C6	PPT at C6 (SD) Pre = 216.1 (103.2) Post = 253.4 (114.2); ES = 0.34 *
						- Nociceptive Flexion Reflex (NFR) threshold	NFR threshold (SD) Pre = 8.0 (5) Post = 7.9 (5.4); ES = -0.02
						- VAS pain from NFR	VAS pain from NFR (SD) Pre = 4.5 (3.8) Post = 3.6 (2.8); ES = 0.27

VAS = visual analogue scale; NVS = numerical verbal scale; HA = headache; avg = average; inter4tile = interquartile; US = ultrasound; NR = not reported; SF-MPQ = Short-form McGill Pain Questionnaire; NPQ = Neck Pain Questionnaire; PPT = pressure pain threshold; ipsi = ipsilateral; contra = contralateral; MTrP's = myofascial trigger points; BP = blood pressure; z-joint = zygapophyseal joint; AROM = active range of motion; EMG = electromyogram.

# # Reported on double placebo registration (not just for sham manipulation)

* Clinically important change

^1^In these two studies, subjects received all treatments; intention-to-treat was not applicable: PEDro score out of 10

### Pain outcomes

Ten (10) trials [15,16,18,26-30,32,34] employed a pain visual analogue scale (VAS) to record neck pain or headache intensity; one [Haas 10] used an 11-point numerical rating scale. Six (6) trials [19-22,25,26], all single-session, employed a pressure threshold algometer to measure pressure pain thresholds over the neck, upper back or upper limb.

### Pain outcomes by control group type

Four trials employed some form of placebo control. Sloop et al.'s trial for neck pain [27] employed anamnestic valium in both groups with the control group receiving no actual manipulations. Outcomes were obtained at an average of two weeks post-treatments. Most patients received only one treatment, while some received two. The mean change in VAS scores in the control group was -5 mm; however, the standard deviation for this value was quite large at 32 mm. Vernon et al.'s [31] trial for tension-type headache employed a factorial design whereby three of four groups received at least one placebo/sham version of the therapies (amitryptiline and spinal manipulation) with one of these groups receiving both placebo treatments. This was the only trial to employ a sham cervical manipulation treatment; however, there was no report of the outcomes for each group separately, so no data were available for this review on the outcome of the double placebo group.

Two trials used de-tuned therapy devices as the control treatment. Tuchin et al.s' [15] multi-session trial for migraine headaches employed de-tuned interferential therapy and reported an average headache intensity reduction in the control group of 17 mm with an effect size of 1.17. Pikula's single session study [32] employed two control groups, one of which received du-tuned ultrasound. The immediate pain reduction averaged 4 mm with an effect size of 0.18.

Using the Sloop et al. and Pikula trials for estimating pain reduction on a VAS in the placebo control groups provides an average of 4.5 mm reduction, which is well below the level most often adopted for minimal clinically important difference and is in accord with the values of placebo control group outcomes reported by Vernon et al. [10] in non-manual therapy trials.

Three trials employed, as the control treatment, cervical manipulation at an "alternate" site. Bakris et al. [33] employed recoil manipulation at what they defined as an ineffective site. As their study investigated the effect of manipulation on blood pressure, no pain-related outcomes were available. They did report virtually no difference in systolic and diastolic blood pressure pre-post intervention in this control group. Pikula [32] employed manipulation to the contralateral side in his single-session control group. He reported an average of 3 mm reduction on a pain VAS (effect size = 0.10). Haas et al. [30] employed a cervical manipulation at an alternate site from the target pain site. This site was determined randomly and compared to sites which had been determined by manual palpation. They reported an average VAS reduction of 16 mm (effect size = 0.78) which is considered a clinically important change.

Data on pain outcomes was available from fourteen (14) trials with non-placebo control groups (5 multi-session; 9 single session). In the five multi-session trials, control groups received either low-level manual contact with no thrust [28,29,34,35] or a waiting list [16]. In three of these studies [16,28,29], the average pain reduction on a VAS was 3 mm. Bove and Nilsson [29] also reported a 7-week follow-up of an average 11 mm reduction in their tension-type headache patient's headache intensity. Haas et al. reported relatively small percentage reductions in headache pain (14.8% for the 8-treatment group, 9.3% for the 16-treatment group at 12 weeks, 15.3% and 9.9% respectively at 24 weeks); however, the effect sizes for these changes were above our threshold (0.90 and 0.52 for 12 weeks; 0.52 and 0.52 for 16 weeks). The fifth trial [34] reported virtually no change in their pediatric headache control group.

Of the nine (9) single-session studies [18-20,22,23,25,26,30,32], two [30,32] employed a contra-lateral or alternate site thrusting manipulation; the other 7 studies used a "manual contact/no thrust" procedure. The mean [95% CI] effect size for VAS reduction of neck pain (five groups [Pikula x2 [32], Haas et al. [30], Martinez-Segura et al. [18], Sterling et al. [26]) was 0.31 [-0.02,0.64]. The mean [95% CI] effect size for neck, trapezius and elbow PPT reduction in 6 groups [19-22,25,26] was 0.34 [0,0.68]. These mean ES just exceeded our threshold for minimally important clinical difference.

Three single-session studies which used manual contact/no thrust controls did not report pain-related outcomes. Their results were as follows: in Buchman et al. [17], the control procedure appeared to have no effect on the presence of palpable cervical segmental dysfunctions; in Tuttle et al. [23], there was no significant change in active ranges of motion; in Dunning and Rushton's trial [24], resting EMG of the biceps muscle increased by an average of 21% on the ipsilateral side and 17% on the opposite side. The first two of these studies provided some support for the proposition that non-thrust procedures do not result in changes in the mobility of the cervical spine.

With regard to clinically important changes, the 21 reviewed studies provided 35 comparisons of baseline to post-treatment pain scores (some trials had no pain-related outcomes; some trials had 2 or 3 comparison times for a pain-related outcome (Haas et al. [35] had 8 such comparisons). In 15 (43%) of these comparisons (8 trials), the control group outcomes exceeded the minimum threshold of 20% reduction of pain/tenderness or effect size greater than 0.30 (See Table 2 for *). In these 8 trials, 5 employed manual contact/no thrust controls [20,21,26,30,35], 1 employed a waiting list [16], 1 employed low level laser and deep massage [29] and 1 employed detuned ultrasound [15]. This latter trial is notable as it was the only one of these 8 trials to use a single-blind placebo treatment; the control group effect size for average headache intensity reduction was 1.17.

The nine (9) multi-session studies merit additional analysis. Seven of these trials [15,16,27-29,34,35 ] provided pain-related outcome comparisons; however, five of them were for headache and only two [16,27] were for neck pain. Within these trials, the control procedures which did not result in a mean reduction of pain that reached the clinically important threshold included anamnestic valium, low power laser + deep frictions, light touch/no thrust.

With respect to the issue of confirming the success of the blinding or the subject's identification of their group assignment, only 3 trials (15%) reported performing this check [27,31,34], all of which used placebo controls. These studies reported that the blinding was generally successful. Two studies which did use a placebo control [32,33] did not report post-intervention registration. None of the studies which used non-placebo control groups reported on the degree to which subjects in each of their study groups could identify their group registration.

## Discussion

The primary objective of this review was to characterize the types of control procedures used in controlled clinical trials of cervical spinal manipulation and the outcomes obtained by subjects in these groups. The goal of this analysis was to identify areas for improvement in future controlled clinical trials. Twenty-one (21) trials were identified, 9 multi-session trials and 12 single-session trials. The most commonly employed control group procedure was "manual contact/no thrust" (12 groups). The clinical outcomes obtained in these control groups are varied, as discussed below.

Clinical trial theory posits that the ideal control treatment should account for all of the non-specific effects of the index treatment but carry none of the direct therapeutic benefits [5-11]. Machado et al. [11] used the following terms to describe these attributes: a placebo treatment that has no known or substantiated therapeutic mechanism is termed "inert"; an inert placebo which mimics the index treatment in all aspects, including the replication of any common side effects is termed "indistinguishable"; when placebos cannot be made indistinguishable, researchers should strive to create "structural equivalence". This term refers to the degree to which the control procedures are as similar in nature and delivery as possible to the index treatment.

Any difference between the index and control treatments that is obtained in the trial ("the trial effect size") should, theoretically, result from the therapeutic mechanism purported to exist in the index therapy. Any deviation from this ideal circumstance has important effects on the potential success of a clinical trial by, for example, increasing the therapeutic effect of the control treatment, thus reducing the trial effect size (Type II error) or by increasing the therapeutic effect of the index treatment (Type I error).

Hawk and colleagues [7-9] and Hancock et al. [5] have noted that the development of placebo manipulation procedures by researchers in manipulative therapy has been challenging. They identified two important objectives of placebo manipulation procedures: 1) the equalization of the non-specific effect of physical touch between groups of subjects, and 2) the blinding of the subject as to the nature of the treatment. Hawk et al. identified the essence of such a placebo manoeuvre in that it "increase(es) the believability of the intervention, thus equalizing the effect of expectation of improvement between groups" [7].

Strong placebogenic effects of manipulation [4-9,36] have been hypothesized. Key factors in this regard include the encouragement of patient relaxation in order to facilitate the procedure, the generic or non-specific effects of manual contact, including fulfillment of patients' expectations regarding manual contact on subjectively felt problem sites and the effect of the thrust and cavitation in fulfilling patient expectations that "something important has just happened". This latter point is often reinforced or amplified by positive feedback statement or behavior from the clinician [36].

Sham cervical manipulation procedures should account for the following issues: tactile contact with the skin, head and neck motions involved in the procedure, mechanical loads applied to the tissues and the sounds associated with them. Differences between a sham and an actual procedure for any one or more of these characteristics might be responsible for cuing the patient as to the nature of the procedure applied. These criteria can be applied to an evaluation of the control procedures described above.

Aside from the control procedure used in Vernon et al., [6,31], which did not involve an actual thrusting manipulation, all other control procedures identified in this review do not provide for the following important elements: a) simulated manual thrust, b) distracting noise to create ambiguity on the issue of cavitation and, c) proven lack of therapeutic effectiveness *a priori*. This combination creates the maximum level of "indistinguishability" possible in manual therapy research. However, with Vernon et al.'s clinical trial [31], the results of the sham manipulation as a control procedure cannot be confirmed separately from the placebo medication, as both were used in the true control group (see below).

The few studies that did employ a single thrusting manipulation control procedure did so on the basis of applying it to sites designated by the investigators as alternative to the "clinically important manipulation site". Whether this was at an alternate segment in the cervical spine [30,32] or at a supposedly non-effective site at the same segment (Bakris et al. [33] who also used a supposedly ineffective thrust direction), by actually providing a "real" manipulation (but at a supposedly inert site), these procedures did not accomplish the goals of simulating thrust and cavitation sounds (they actually produced them). Furthermore, these procedures were not tested previously for their inertness; in other words, there may have been some element of "indistinguishability", but the level of inertness was not established *a priori.*

In the case of Haas et al. [30], the alternate site procedure produced clinically important changes that were roughly equivalent to the index procedure; thus invalidating this procedure as a useful control manipulation (although the intention of these authors was not to establish the alternate site approach as a "control" procedure). In the case of both Pikula [32] and Bakris et al. [33], their control groups' results were only confirmed posteriorly. Pikula's small study provides very limited support for the idea that a real manipulation at a site designated as clinically less important may work as a control procedure in a single session. In the case of Bakris et al., it appears that their control procedure is highly dependent on the model of manipulation and the skill of the chiropractor involved and may not be generalizable to other circumstances.

Distinguishing control procedures which do and don't employ thrusts becomes important for two reasons. These will be discussed with respect to manual-type procedures and then non-manual type procedures. With respect to manual-type procedures, the first issue pertains to the control group subjects who receive non-thrust procedures, particularly those that involve manual contact without thrust (the predominant category in this review). While the strategy of "manual contact without thrust" does account for some similarities with real manipulation in patient positioning and in initial manual contact, such subjects do come to know after-the-fact that they have not received a thrusting procedure (because there is no simulation of thrust and cavitation noise). This may become incongruent with their expectations, especially in multi-session trials, and create a psychological factor superimposed on the more direct treatment-related outcome (which should, theoretically, be minimal). This could even rise to the status of a "nocebo" effect if the subject's posterior knowledge and resultant unmet expectations (especially over several sessions) combine to result in a negative attitude to the circumstances and in a poorer response on clinical outcome measures.

On the other hand, from Table 3 it can be seen that, in single session studies using manual contact/no thrust procedures, most often the control groups reported no significant or clinically important changes in pain, tenderness or other singular physical findings. Despite the issues raised above on lack of equivalence and the effects of unmasking, this type of procedure may be satisfactory for single session studies of the immediate effects of cervical manipulation. Given the fact that, in a small number of studies, clinically important changes in pain or tenderness were reported, it is advisable that researchers conduct a pre-test of this procedure in their hands to insure that it is generally inert before using it in a larger randomized trial.

**Table 3:** Summary of single-session studies using manual contact/no thrust control procedure

Buchman et al. [17]	Single session	No change in fixations
Martinez-Segura et al. [18]	Single session	No change in neck pain

Fernandez De Las Penas et al. [19]	Single session	No change in elbow PPT

Ruis-Saez et al. [20]	Single session	Moderate change trapezius TP at 5 and 10 minutes follow-up

Fernandez-Carnero et al. [21]	Single session	No change in elbow PPT

Fernandez De Las Penas et al. [22]	Single session	No change in neck PPT

Tuttle [23]	Single session	No change in ROM

Dunning and Rushton [24]	Single session	Moderate change in biceps EMG

Mansila-Ferragut et al. [25]	Single session	No change in TMJ PPT

Sterling et al. [26]	Single session	Moderate change in Neck PPT

The second issue applies to those subjects who receive the "real manipulation" in studies where other groups of subjects do not. By experiencing thrusting manipulations (with resultant audible cavitations (i.e., clicking sounds), subjects in these groups automatically receive indications which they would interpret to mean that they did receive the "index" treatment. This may result in an effect opposite to the one described above, where their treatment expectations are strongly confirmed. This may result in a non-specific effect which adds to that of the direct effects of the therapy, leading to a positive augmentation of the clinical outcomes (especially those that require subjective responses, and especially those related to satisfaction ratings). In passing, it should be remarked that in virtually all prior clinical trials of manipulation for neck pain and headaches, this is the situation that prevails in the groups receiving spinal manipulation.

With regard to non-manual control procedures, these do more readily lend themselves to the creation of placebo versions by, for example, de-tuning the equipment or applying very low doses of therapy. However, these procedures account for none of the manipulation-specific issues discussed above, making comparisons between these groups problematic, especially when issues of "mechanism of action" become important for the investigation. On strictly pragmatic grounds, non-manual placebo control procedures (such as those reviewed in Vernon et al. [10]) may be satisfactory for manipulation trials as they clearly result in clinical outcomes below the threshold of minimal clinically important difference.

With the exception of Vernon et al. [31], all studies have employed a single control procedure. Even if a single procedure is somewhat successful at masking subjects, it must do so entirely on its own. The "double placebo technique" [37-41] uses two procedures (either in a factorial design or in a simpler 2 or 3-group design) to increase the effectiveness of masking. While there is some evidence from the trial by Vernon et al. that this strategy was successful, more studies on this approach are needed.

It is difficult to summarize the clinical outcomes of the control groups analysed in this review on account of the highly variable methods and results. In 43% of the pain-related comparisons used in approximately one-third of the trials (8/21), the control procedure resulted in mean changes (reductions) that would be deemed clinically important. On the other hand, in some proportion of each of the categories of control procedures, these procedures resulted in mean changes which were below the minimal clinically important threshold.

With regard to the multi-session studies, where three trials did report mean changes in the control groups that did not exceed the minimal clinically important threshold, several caveats are offered. In the case of Sloop et al. [27], while the control group receiving anamnestic valium + no manipulation reported very little improvement, most of the subjects in that study received only one treatment session and the outcome was obtained two weeks following treatment. In the case of Borusiak et al. [34], it should be noted that this trial, which employed a light touch/no thrust control procedure, included only children as subjects; this finding makes it difficult to justify the use of this control procedure in multi-session clinical trials with adults.

Beyond the issues of indistinguishability, equivalence and clinical effect, a very serious issue is the lack of determination of the level of blinding in the vast majority of these studies. In many cases, a result in favor of the manipulation group (especially in single-session studies) has been superficially interpreted to mean that a beneficial effect exists for manipulation, when, in fact, the investigators have no idea as to the degree to which subjects in the control group(s) may have become unblinded and how that may have affected their responses. There is a strong possibility of a Type I error which is then ignored. This problem is not confined to spinal manipulation as a therapy nor is it confined to treatments for the neck alone. Machado et al. [11] and Puhl et al. [42] have shown that the same problem exists for other pharmacologic and non-pharmacologic treatments for low back pain.

Finally, for single-session studies of clinical efficacy, every attempt should be made to report on randomization concealment and intention-to-treat, as this would make for more complete reporting and easier quality scoring.

This review has limitations. The entry-level search strategy was more broadly defined in order to identify the largest range of potential studies. While a search strategy with a more highly specified algorithm might have been employed, we are confident that our strategy is ultimately replicable by other investigators.

We did not specify the results of each item of the PEDro scale for each study, as all studies achieved a score above our threshold for acceptability. In this review, we felt that such an analysis, typically found in other systematic reviews, was not necessary, as the primary objective was not a methodological review of the studies themselves, but of the control group procedures used.

As noted in the Methods, pooling of outcomes data was not conducted. This was due to several factors. First, contrary to other systematic reviews, it was not our intention to derive a pooled estimate of the effect of a specific treatment on specific clinical entities; rather, we sought to characterize the various control group procedures used in a broad range of studies whose primary objective was the determination of the treatment effect of the index treatment, i.e., spinal manipulation. This lead to our review including a wide variety of studies with considerable heterogeneity on issues of clinical condition, subjects selected, outcomes measures and end points used, etc.

This heterogeneity made the reporting of outcomes more difficult, and less conventional, leading to our narrative account of the results.

## Conclusion

The most commonly used control group procedure in clinical trials of cervical manipulation is manual contact/no thrust. Most control procedures in cervical manipulation trials do not result in subject blinding. Clinical outcomes of these groups were varied with about one third of groups demonstrating a clinically important change. The greatest majority of trials do not report on post-intervention blinding. The effect of unmasking of control subjects makes the interpretation of the existing clinical trials challenging. At the very least, future clinical trial reports should include an indication of the post-intervention registration of group allocation by subjects. A small number of candidate procedures exists for effective control interventions which create optimum combinations of inertness and practical equivalence with real manipulative therapies. Much more research is required to improve this important aspect of clinical trial methodology in cervical manipulation studies.

## Competing interests

The authors declare that they have no competing interests.

## Authors' contributions

HV was responsible for following: the design of the study, selection of the studies, data collection and analysis, preparation of the manuscript.

AP was responsible for: scoring all studies, data extraction and preparation of the manuscript.

CR was responsible for: scoring all studies, data extraction and assistance with the preparation of the manuscript.

All authors read and approved the final manuscript.
